# Remimazolam-based total intravenous anesthesia in a patient with a confirmed diagnosis of malignant hyperthermia: a case report

**DOI:** 10.1186/s40981-024-00710-7

**Published:** 2024-04-22

**Authors:** Hiroshi Kondo, Keiko Mukaida, Kurumi Sasai, Yukiko Nao, Ken Hashimoto, Hirotsugu Miyoshi, Rieko Kanzaki, Yasuo M. Tsutsumi

**Affiliations:** 1 Department of Anesthesiology, National Hospital Organization Higashihiroshima Medical Center, Higashihiroshima, Japan; 2 Department of Anesthesiology and Critical Care, Hiroshima University, Hiroshima, 734-8551 Japan

**Keywords:** Malignant hyperthermia, Ryanodine receptor1, Remimazolam

## Abstract

**Background:**

Malignant hyperthermia (MH) is a rare, life-threatening disorder of calcium homeostasis in skeletal muscle cells that is triggered by volatile anesthetics and succinylcholine, leading to a hypermetabolic reaction. The pathogenic ryanodine receptor 1 (RYR1) gene variant is critical. Patients susceptible to MH should avoid triggering agents, and total intravenous anesthesia (TIVA) is preferred. Remimazolam is safe in patients with suspected MH.

**Case presentation:**

We present the first case of remimazolam treatment in a genetically confirmed patient with MH without MH development. A 72-year-old man with a family history of MH underwent remimazolam-based TIVA. After informed consent was obtained, a muscle biopsy and genetic testing were performed. Intraoperatively and postoperatively, the patient exhibited no signs of MH. An enhanced function of the RYR1 channel into releasing calcium was indicated, and the genetic testing revealed a pathogenic variant of *RYR1*.

**Conclusions:**

Remimazolam-based TIVA is safe in patients confirming the diagnosis of MH.

## Background

Malignant hyperthermia (MH) is a rare, life-threatening, and pharmacogenetic disorder precipitated by the administration of halogenated volatile anesthetics, such as sevoflurane or desflurane and the depolarizing muscle relaxant succinylcholine [1–3]. MH crisis manifests as a hypermetabolic reaction triggered by an uncontrolled surge in calcium levels within skeletal muscle cells induced by these agents. Key players in MH pathogenesis include ryanodine receptor 1 (RYR1), situated on the sarcoplasmic reticulum (SR) membrane, and the voltage-dependent calcium channel (Cav1.1) embedded in the muscle cell membrane.

Patients susceptible to MH should strictly avoid exposure to triggering agents [4, 5]. Total intravenous anesthesia (TIVA) is generally preferred for these individuals [4, 5]. Remimazolam, a short-acting benzodiazepine, is used for both the induction and maintenance of general anesthesia in Japan and represents a choice for TIVA [6]. We have demonstrated in cellular studies that remimazolam does not increase intracellular calcium concentrations in the range of concentrations for clinical use in RYR1 mutant cells, which cause MH [7, 8]. Moreover, remimazolam has been reported safe for use in patients with suspected MH [9–11], but there remains a lack of case reports on using remimazolam in patients with a definitive diagnosis of MH. Here, we present the first case report of genetically confirmed MH in which remimazolam was used for TIVA without the development of MH.

## Case presentation

Written informed consent was obtained from the patient to publish this case report.

A 72-year-old man (height, 1.61 m; weight, 71 kg) was admitted for scheduled endoscopic sinus surgery and nasal septoplasty to treat chronic sinusitis. He had previously undergone four surgical procedures under general anesthesia without any adverse events. Two general anesthesia events occurred at another hospital, while the remaining two surgeries were performed under total intravenous anesthesia (remifentanil and propofol). The patient routinely took medication for hypertension. Preanesthetic examination and laboratory evaluation results were within the normal range, except for a mild increase in serum creatine kinase (CK) level of 599 IU/L (reference range: ≤ 240 IU/L for men).

The patient had a family history of MH. His son died at the age of 5 from an MH crisis during general anesthesia with enflurane and succinylcholine 30 years before. His son experienced unexplained tachycardia, tachypnea, muscle rigidity, and elevated body temperatures. The MH crisis led to a cardiac arrest, resulting in death 3 h after induction.

We recommended the patient to undergo a muscle biopsy for calcium-induced calcium release (CICR) testing concurrent with his scheduled surgery and phlebotomy for genetic analysis. Informed consent was obtained from the patient for CICR and genetic testing.

**Fig. 1:**
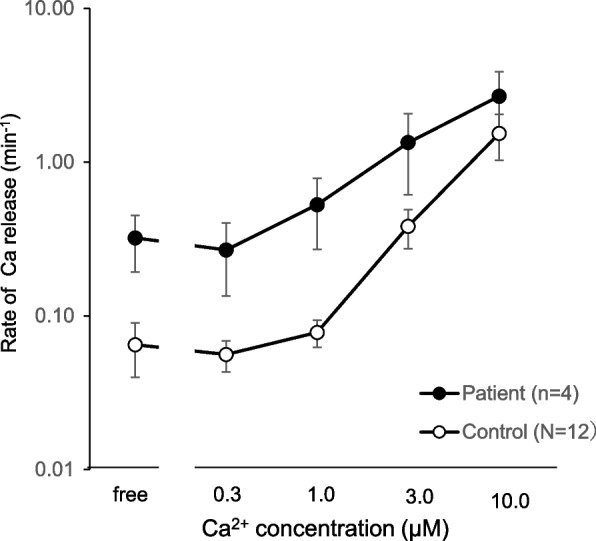
Ca-induced Ca release (CICR) test results. The patient showed a clear acceleration of the CICR rate from the SR compared with the control. The control group consisted of 12 MH-negative individuals diagnosed using in vitro and caffeine-halothane contracture tests. Data are expressed as mean ± SD. *N* indicated the number of cases; *n* indicated the number of measurements in this patient

The anesthetic workstation was prepared in accordance with the recommendations of the European Malignant Hyperthermia Group (EMHG) [7]. Initial doses of dantrolene and distilled water were kept on hand so that they could be administered immediately upon the appearance of signs of MH. Our standard monitors, including noninvasive blood pressure, electrocardiogram, capnography, pulse oximetry, and bispectral index (BIS), were initiated along with a body core temperature monitor (3 M Japan Co., Ltd.). The body temperature at the start of anesthesia was 36.1 °C. General anesthesia was induced with intravenous remimazolam 0.1 mg/kg, fentanyl 50 μg, and rocuronium 50 mg. The intraoperative ventilation mode had a tidal volume of 480 mL, a respiratory rate of 12 breaths/minute, and an inspiratory to expiratory (I:E) ratio of 1:2. After induction of anesthesia, the end-expiratory carbon dioxide pressure (EtCO_2_) was 34.1 mmHg. General anesthesia was maintained with continuous administration of remimazolam and remifentanil. The remimazolam dose rate was adjusted according to the BIS values. Rocuronium was administered at a dose of 10 mg using a muscle-relaxation monitor. At the end of the surgery, the patient’s temperature was 36.0 °C, and EtCO_2_ was 33.7 mmHg. Surgery, including muscle biopsy, was completed without complications, such as an MH crisis or severe hypotension. The patient spontaneously awakened from anesthesia without antagonists (sugammadex or flumazenil) within 10 min after completion of surgery. The train-of-four ratio was greater than 90%, and the tidal volume was approximately 500 mL. The patient was then extubated. Before returning to the general ward, flumazenil 0.2 mg was administered to achieve complete awakening. He had an uneventful postanesthesia period without high fever, myalgia, or dark urine. CK level was slightly elevated to 786 IU/L on postoperative day 1. The CICR rate was measured using chemically skinned muscle fibers, according to the Endo method [12]. The CICR rates were higher than the controls (Fig. 1). This result indicates a predisposition to MH due to the enhanced function of the RYR1 channel in releasing calcium from the SR.

**Fig. 2:**
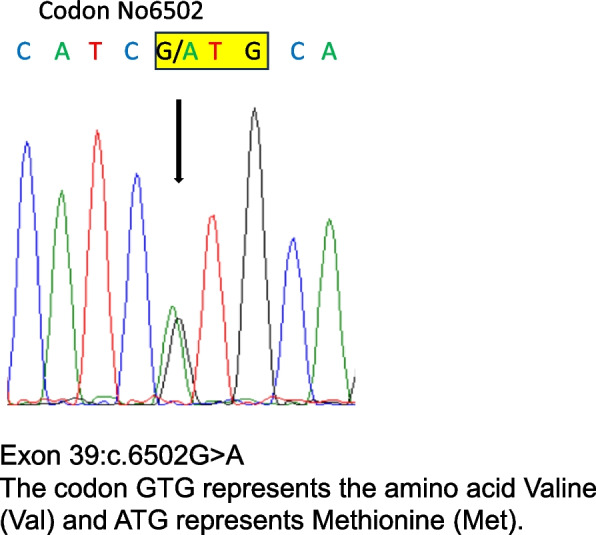
Sanger sequencing results. Electropherogram obtained by Sanger sequencing of *RYR1* exon 39 showing a region between codons 6498 and 6506. The 6502nd codon was a mixture of G and A (c.6502G > A), indicating that it was a heterozygous missense variant with amino acid change (valine → methionine: p.Val2168Met). This variant was previously described as pathogenic in ACMG/AMP and EMHG

Genetic testing was performed using whole-exome sequencing with panel analysis of 24 genes associated with MH, including *RYR1* and *CACNA1S*. A heterozygous *RYR1* variant was identified in exon 39 (c.6502G > A, p. Val2168Met). This missense variant was confirmed using Sanger sequencing (Fig. 2). This variant has been described as a pathogenic variant associated with MH by the American Society of Clinical Genetics and Genomics and Molecular Pathology Society (ACMG/AMP) [2, 5] and EMHG (https://www.emhg.org/diagnostic-mutations). No other relevant variants were detected in *RYR1* or different genes.

Based on the results of both tests, we conclusively diagnosed the patient with MH predisposition.

## Discussion

In general, TIVA with propofol is provided as “trigger-free” anesthesia for MH-susceptible patients. We chose remimazolam-based TIVA for general anesthesia in our patient suspected MH. The patient experienced intra- and postoperative period without developing MH or other adverse events. The successful administration of remimazolam-based TIVA in our MH patient with a confirmed diagnosis of MH underscores its potential as a “trigger-free” anesthetic option.

Remimazolam is a short-acting benzodiazepine rapidly hydrolyzed by tissue esterases. Midazolam, a benzodiazepine, as well as remimazolam, has demonstrated safety in MH-predisposed individuals [13], and its suitability in this context has been less explored. Remimazolam, similar to midazolam, may be considered safe for patients with malignant hyperthermia. Basic cellular experiments have shown that remimazolam does not enhance Ca^2+^ elevation in human embryonic kidney (HEK-293) cells expressing mutant-type ryanodine receptor *RYR1* [7]. Although exposure to remimazolam increased Ca^2+^ levels in myotubes cultured from CICR-accelerated patients, like this case, the concentration of remimazolam was reported to be 80 times higher than the clinical concentration [8]. In addition, a few clinical cases have been reported in which remimazolam was safely used under general anesthesia in patients with suspected MH [9–11]. However, these cases were not definitively diagnosed as MH. To the best of our knowledge, this is the first report of the safe use of remimazolam in a patient with a confirmed diagnosis of MH using both genetic and CICR testing.

Moreover, remimazolam-based TIVA has been reported to have a milder effect on cardiac contractility than propofol-based TIVA, and patients treated with remimazolam are less likely to develop severe hypotension [14]. Remimazolam does not cause the infusion pain experienced with propofol and can be reversed with flumazenil. It can be used in patients with elevated CK levels, as in this case, without causing side effects, such as propofol infusion syndrome or rhabdomyolysis [15].

MH is an autosomal-dominant genetic disease of* RYR1*, and *CACNA1S* encodes the subunit alpha1 S of Cav1.1. DNA screening, muscle contracture testing, or both are recommended as first-line diagnostic tests for patients with suspected MH [3]. DNA analyses of peripheral venous blood samples are less invasive than muscle contracture tests that require muscle biopsy, but the probability of detecting disease-causing variants is low, at 30 to 50% [16]. *RYR1* and *CACNA1S* variants are associated with MH and diverse forms of congenital myopathy [3]. In addition, among the over 400 reported *RYR1* variants linked to MH [16], only those with a gain in calcium release function (increased sensitivity to RYR1 agonists) are pathogenic [2, 16, 17]. The variant curation expert panel of ACMG/AMP reported that *RYR1* variants classified as pathologic and likely pathogenic comprised a quarter of the 335 MH-related variants, and two-thirds were classified as variants of uncertain significance (VUS) [2]. Despite negative DNA test results, predisposition cannot be ruled out [2, 3, 16]. Therefore, testing with muscle biopsy is the next step in diagnosis [3]. If variants suspected to be associated with MH are found and considered variants of VUS, further study of the Ca^2+^-regulating function of these variants is needed [2, 16, 17]. In addition, there is some discordance between the results of MH genetic analysis and diagnostic muscle contraction testing [18, 19]. In such cases, we recommend genetic analysis and CICR testing. The patient agreed to undergo two tests. Both tests revealed a pathogenic variant (p.Val2168Met) in *RYR1* and the enhanced function of the RYR1 channel into releasing calcium from SR.

In conclusion, our study highlights the feasibility and safety of remimazolam-based TIVA in MH-prone patients. Further research and clinical validation are warranted to confirm the role of remimazolam in eliminating the risk of MH development during anesthesia.

## Data Availability

However, this is not applicable because of concerns about patient privacy.

## References

[1] Riazi S, Kraeva N, Hopkins PM (2018). Updated guide for the management of malignant hyperthermia. Can J Anaesth.

[2] Johnston JJ, Dirksen RT, Girard T, Hopkins PM, Kraeva N, Ognoon M (2022). Updated variant curation expert panel criteria and pathogenicity classifications for 251 variants for RYR1-related malignant hyperthermia susceptibility. Hum Mol Genet.

[3] Frassanito L, Sbaraglia F, Piersanti A, Vassalli F, Lucente M, Filetici N (2023). Real evidence and misconceptions about malignant hyperthermia in children: a narrative review. J Clin Med.

[4] Hopkins PM, Girard T, Dalay S, Jenkins B, Thacker A, Patteril M (2021). Malignant hyperthermia 2020: guideline from the Association of Anaesthetists. Anaesthesia.

[5] Rüffert H, Bastian B, Bendixen D, Girard T, Heiderich S, Hellblom A (2021). Consensus guidelines on perioperative management of malignant hyperthermia suspected or susceptible patients from the European Malignant Hyperthermia Group. Br J Anaesth.

[6] Hirota K (2023). Remimazolam: a new string to the TIVA bow. J Anesth.

[7] Watanabe T, Miyoshi H, Noda Y, Narasaki S, Morio A, Toyota Y (2021). Effects of remimazolam and propofol on Ca(2+) regulation by ryanodine receptor 1 with malignant hyperthermia mutation. BioMed Res Int.

[8] Miyoshi H, Watanabe T, Kido K, Kamiya S, Otsuki S, Narasaki S (2022). Remimazolam requires less vasopressor support during induction and maintenance of general anesthesia in patients with severe aortic stenosis undergoing transcatheter aortic valve replacement: a retrospective analysis from a Single Center. BioMed Res Int.

[9] Uchiyama K, Sunaga H, Katori N, Uezono S (2021). General anesthesia with remimazolam in a patient with clinically suspected malignant hyperthermia. JA Clin Rep.

[10] Matsumoto T, Sakurai K, Takahashi K, Kawamoto S (2022). Use of remimazolam in living donor liver transplantation: a case report. JA Clin Rep.

[11] Petkus H, Willer BL, Tobias JD (2022). Remimazolam in a pediatric patient with a suspected family history of malignant hyperthermia. J Med Cases..

[12] Ohta T, Endo M, Nakano T, Morohoshi Y, Wanikawa K, Ohga A (1989). Ca-induced Ca release in malignant hyperthermia-susceptible pig skeletal muscle. Am J Physiol..

[13] Fletcher JE, Rosenberg H, Hilf M (1984). Effects of midazolam on directly stimulated muscle biopsies from control and malignant hyperthermia positive patients. Can Anaesth Soc J.

[14] He M, Gong C, Chen Y, Chen R, Qian Y (2023). Effect of remimazolam vs. propofol on hemodynamics during general anesthesia induction in elderly patients: single center, randomized controlled trial. J Biomed Res..

[15] Kempenaers S, Hansen TG, Van de Velde M (2023). Remimazolam and serious adverse events: a scoping review. Eur J Anaesthesiol.

[16] Hoppe K, Jurkat-Rott K, Kranepuhl S, Wearing S, Heiderich S, Merlak S (2021). Relevance of pathogenicity prediction tools in human RYR1 variants of unknown significance. Sci Rep.

[17] Johnston JJ, Dirksen RT, Girard T, Gonsalves SG, Hopkins PM, Riazi S (2021). Variant curation expert panel recommendations for RYR1 pathogenicity classifications in malignant hyperthermia susceptibility. Genet Med.

[18] Ibarra Moreno CA, Kraeva N, Zvaritch E, Figueroa L, Rios E, Biesecker L (2020). A multi-dimensional analysis of genotype-phenotype discordance in malignant hyperthermia susceptibility. Br J Anaesth.

[19] Noda Y, Miyoshi H, Benucci S, Gonzalez A, Bandschapp O, Girard T (2023). Functional characterization of RYR1 variants identified in malignant hyperthermia susceptible individuals. Neuromuscul Disord.

